# Low Abundance of Circulating Tumor DNA in Localized Prostate Cancer

**DOI:** 10.1200/PO.19.00176

**Published:** 2019-09-09

**Authors:** S. Thomas Hennigan, Shana Y. Trostel, Nicholas T. Terrigino, Olga S. Voznesensky, Rachel J. Schaefer, Nichelle C. Whitlock, Scott Wilkinson, Nicole V. Carrabba, Rayann Atway, Steven Shema, Ross Lake, Amalia R. Sweet, David J. Einstein, Fatima Karzai, James L. Gulley, Peter Chang, Glenn J. Bubley, Steven P. Balk, Huihui Ye, Adam G. Sowalsky

**Affiliations:** ^1^ National Institutes of Health, Bethesda, MD; ^2^ Beth Israel Deaconess Medical Center, Boston, MA

## Abstract

**PURPOSE:**

Despite decreased screening-based detection of clinically insignificant tumors, most diagnosed prostate cancers are still indolent, indicating a need for better strategies for detection of clinically significant disease before treatment. We hypothesized that patients with detectable circulating tumor DNA (ctDNA) were more likely to harbor aggressive disease.

**METHODS:**

We applied ultra-low-pass whole-genome sequencing to profile cell-free DNA from 112 patients diagnosed with localized prostate cancer and performed targeted resequencing of plasma DNA for somatic mutations previously identified in matched solid tumor in nine cases. We also performed similar analyses of data from patients with metastatic prostate cancer.

**RESULTS:**

In all cases of localized prostate cancer, even in clinically high-risk patients who subsequently had recurrent disease, ultra-low-pass whole-genome sequencing and targeted resequencing did not detect ctDNA in plasma acquired before surgery or before recurrence. In contrast, using both approaches, ctDNA was detected in patients with metastatic prostate cancer.

**CONCLUSION:**

Our findings demonstrate clear differences between localized and advanced prostate cancer with respect to the dissemination and detectability of ctDNA. Because allele-specific alterations in ctDNA are below the threshold for detection in localized prostate cancer, other approaches to identify cell-free nucleic acids of tumor origin may demonstrate better specificity for aggressive disease.

## INTRODUCTION

During the past two decades, prostate cancer has remained the most diagnosed neoplasm in American men, representing approximately 20% of all new diagnoses in 2019.^1^ Overtreatment of newly diagnosed, indolent prostate cancers detected by increasing levels of prostate-specific antigen (PSA) has been mitigated by increasingly widespread adoption of active surveillance, magnetic resonance imaging–targeted biopsies, nomograms, and molecular tests for assessing the risk posed by unsampled higher-grade disease.^2-5^ Although the absence of adverse pathologic features, such as high Gleason score or seminal vesicle invasion, from a biopsy specimen is associated with improved outcomes after definitive therapy (ie, surgery or radiation), sampling errors may lead to underestimation of the risk of biochemical recurrence. The potential for failure to detect pathologic features motivates increased biopsy frequency and premature withdrawal from active surveillance.^6-8^

Numerous recent studies have explored the genomic basis for development of localized prostate cancer, showing distinct evolutionary paths in nonindolent versus indolent disease. The fate of tumors to progress from their somatic progenitors is set early, with alterations in *ATM*, *PTEN*, and *MYC* having predictive power for the existence of higher-grade disease, including occult oligometastases, at the time of radical prostatectomy.^9-13^ The vast majority of these alterations occur as copy number gains or deletions; thus, the percentage of the genome affected by large chromosomal rearrangements is similarly predictive of biochemical recurrence and poor outcome.^10,14,15^

Analysis of plasma cell-free DNA (cfDNA) has rapidly gained traction for profiling tumor genomics in patients with metastatic disease, especially in prostate cancer, in which dissemination to the bone occurs frequently.^16^ Allele-specific assays that detect major driver events, such as mutations to *AR*, *APC*, *EGFR*, and *ERBB2* are commercially available for identification of recurrent, targetable clonal alterations in advanced stages of several cancers, including prostate, colorectal, lung, and breast cancer.^17^ Comprehensive cancer panels, as well as whole-genome and -exome sequencing, can also be used to interrogate somatic copy number alterations (SCNAs) from plasma DNA, with varying resolution depending on the sequence modality and depth.^18,19^ Personalized sequencing assays have shown sensitivity for the detection of urothelial and colorectal cancers.^20,21^ The success of these approaches has been thought to depend on high tumor burden and the propensity of the tumor to shed circulating tumor DNA (ctDNA) into the bloodstream with proportional contribution of subclones to the ctDNA pool.^22,23^ However, the feasibility of applying these approaches to assess the clinical trajectory of patients with newly diagnosed prostate cancer has not been established.

**Context**

**Key Objective**
We evaluated whether detection of circulating tumor DNA (ctDNA) in patients with localized prostate cancer was associated with high-grade pathologic features and biochemical recurrence. We examined ctDNA detection using unbiased ultra-low-pass whole-genome sequencing approaches as well as focused resequencing on the basis of multiregion sampling from radical prostatectomy tissue.
**Knowledge Generated**
ctDNA was not detectable in plasma from patients before radical prostatectomy. In patients who subsequently experienced biochemical recurrence, ctDNA was not detectable in serially collected plasma over 24 months after surgery, including a patient who had disease recurrence.
**Relevance**
DNA-based liquid biopsy approaches for localized prostate cancer are hampered by the low overall abundance of ctDNA. Although these methods work well in metastatic disease, prostate-specific antigen remains the gold standard for sensitivity for diagnosing recurrence after primary therapy.

In this study, we performed ultra-low-pass (ULP) whole-genome sequencing (WGS) of cfDNA from 112 patients with localized prostate cancer to assess genome-wide SCNAs and their association with biochemical recurrence-free survival (median follow-up, 50 months). We also performed deeper, targeted sequencing of cfDNA in nine cases with matched multiregion sequencing of prostate tumor tissue to identify subclones in ctDNA that may associate with adverse pathologic features or mediate relapse. The absence of signal from ctDNA in plasma from patients with localized, but not metastatic, prostate cancer demonstrates that the strategy of using tumor-specific somatic alterations for assessing disease burden is of minimal clinical utility.

## METHODS AND RESULTS

### Large SCNA Events Were Not Detectable in the Plasma of Patients With Localized Prostate Cancer

ULP-WGS has been proposed as a screening technique to detect large SCNAs in cfDNA for the rapid and inexpensive determination of ctDNA content.^19^ To assess the feasibility of this analysis in patients with localized disease, blood was obtained from 112 consecutive patients (case numbers L001 to L112) between April 2014 and January 2016. Patients consented to participate in tissue and blood procurement protocols while undergoing radical prostatectomy (RP) as definitive therapy for newly diagnosed prostate cancer or previously diagnosed prostate cancer that had progressed on active surveillance. Clinical demographics for this cohort are given in Table 1. Blood was collected from an additional seven consecutive patients (case numbers M01 to M07) with radiographically confirmed metastatic prostate cancer who would be expected to harbor ctDNA on the basis of high tumor volumes (Table 1).

**TABLE 1.**
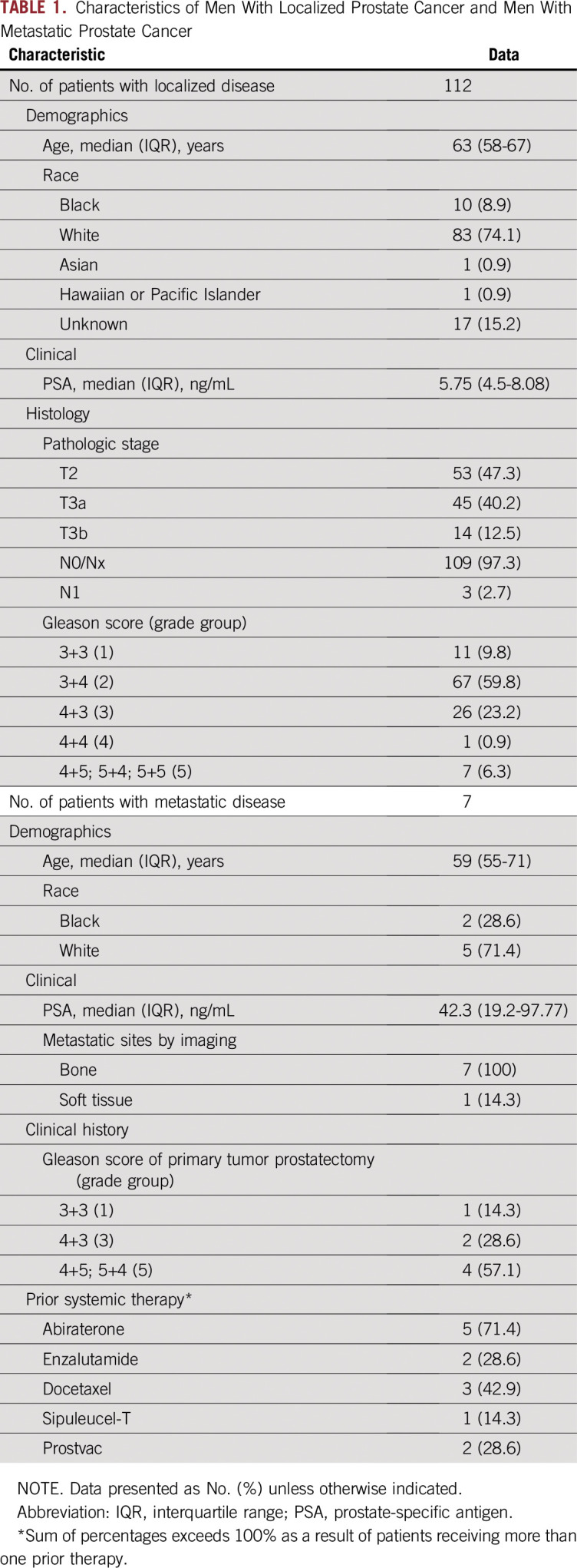
Characteristics of Men With Localized Prostate Cancer and Men With Metastatic Prostate Cancer

**FIG 1.**
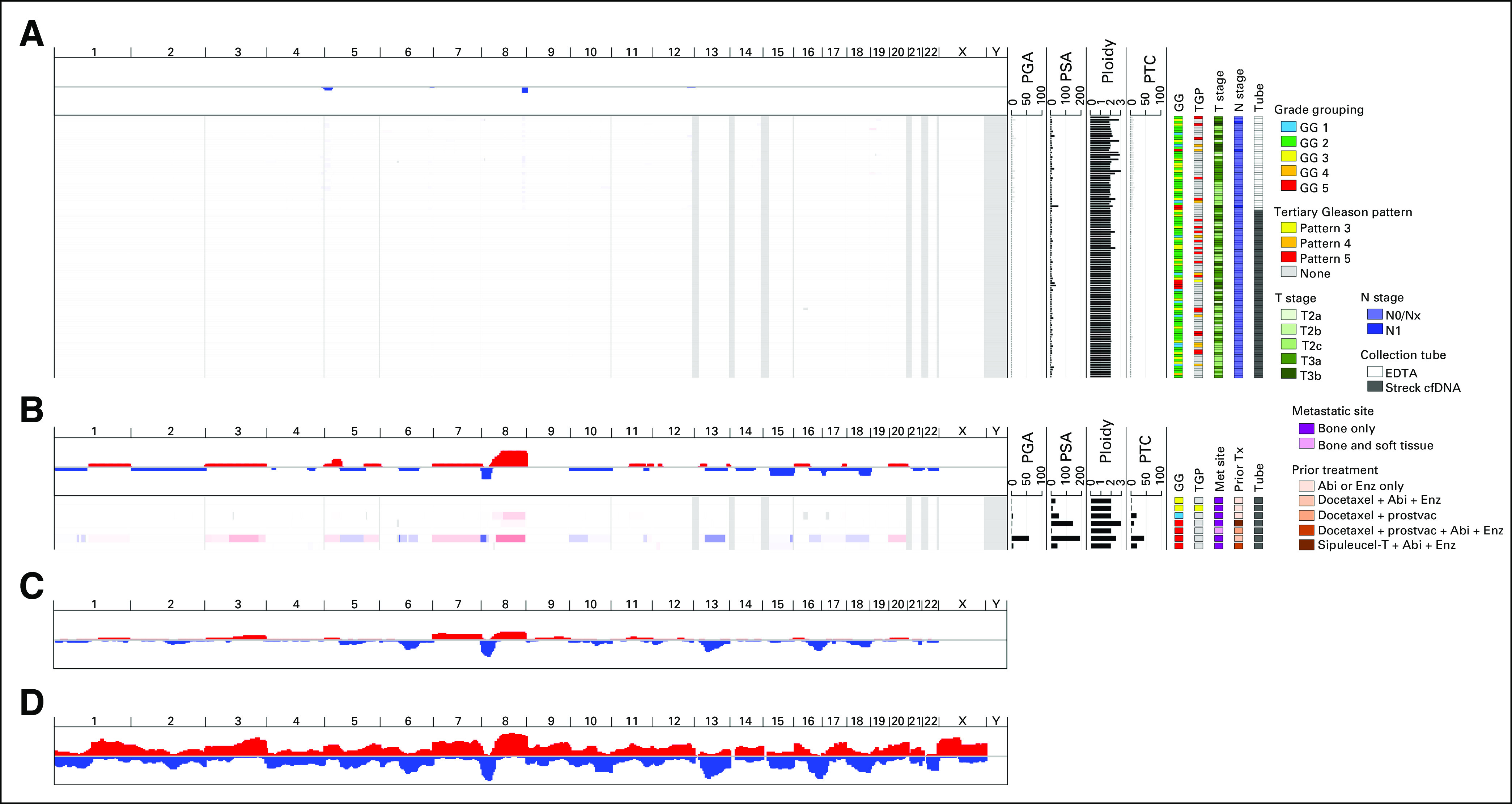
Ultra-low-pass whole-genome sequencing of circulating tumor DNA. (A) Somatic copy number alteration (SCNA) profile of circulating tumor DNA (ctDNA) from patients with localized prostate cancer, encompassing National Comprehensive Cancer Network risk groups of low, intermediate-favorable, intermediate-unfavorable, high, and very high-risk disease (n = 112). Gray bars represent PGA and PTC values before artifact removal. Ploidy values are uncorrected. (B) SCNA profile of ctDNA from patients with radiographically confirmed, metastatic, castration-resistant prostate cancer (n = 7). (C) SCNA profile of patients in the prostate The Cancer Genome Atlas^24^ cohort (n = 333). (D) SCNA profile of patients in the Prostate Cancer Foundation-Stand Up to Cancer^25^ cohort (n = 150). Abi, abiraterone acetate plus prednisone; Enz, enzalutamide; GG, International Society of Urological Pathology grade grouping; Met, metastatic; PGA, percent genome altered; PSA, prostate-specific antigen; PTC, percent tumor content; TGP, tertiary Gleason pattern; Tx, therapy.

We performed ULP-WGS on plasma collected before RP in the 112 patients with localized disease (Fig 1A) to an average depth of 0.36× (range, 0.19× to 0.74×). Plasma from the first 40 patients was collected in K_2_-EDTA tubes; the remainder of blood samples were collected in Streck Cell-Free DNA blood collection tubes (BCTs; Streck, La Vista, NE). With the exception of systemic artifacts in chromosomes 5, 6, 8, and 12 from all plasma collected in the EDTA tubes, no SCNAs were detected. Similarly, in the Streck-collected samples, no SCNAs were detected except for random sequencing artifacts in five patients. Because average percent tumor content (PTC) is calculated on the basis of all SCNA events, removal of these artifacts resulted in no calls of PTC. The majority of patients (95 of 112) had PSA levels no higher than 10 ng/mL. Even the patient with the highest PSA level in the entire cohort, 43.63 ng/mL, did not have nonartifactual SCNAs typical of prostate cancer. Consequently, PTC and percent genome altered were indeterminate for the localized cohort.

In contrast, four of the seven patients with metastatic disease had plasma harboring substantial quantities of ctDNA, as detected by ULP-WGS (Fig 1B). The SCNA profile of this cohort was similar to that of The Cancer Genome Atlas prostate cohort (Fig 1C) and even more similar to the metastatic Prostate Cancer Foundation–Stand Up to Cancer cohort (Fig 1D). The patient with the highest PTC (30.66%) had a PSA level of 190.9 ng/mL, and the patient with the lowest detectable PTC (6.8%) had a PSA level of 144.2 ng/mL. In contrast, in metastatic prostate cancer, the lowest PSA level associated with detectable tumor was 42.3 ng/mL, corresponding to 13.94% tumor content. Therefore, we conclude that ULP-WGS is not sensitive for the detection of SCNAs in the plasma of patients with localized prostate cancer and that PSA level in the localized setting is a poor surrogate for likelihood of detecting ctDNA by ULP-WGS.

### Requirements for a Patient-Specific Assay

Development of primary prostate cancer is driven primarily by structural rearrangements and SCNAs; hotspot point mutations in oncogenes and tumor suppressors, such as *HRAS* and *TP53*, are rare.^24^ Commercial, off-the-shelf ctDNA tests are focused on these recurrent mutations, limiting their utility for detecting ctDNA in primary prostate cancer. Even the most recurrent mutation in primary prostate cancer, at codon 133 of *SPOP*, occurs in less than 5% of tumors.^24^ Presuming mutation events that occur early in a tumor’s natural history are present in all daughter cells, truncal passenger mutations would be present in ctDNA and, therefore, might be used to detect ctDNA on a per-patient basis.

**FIG 2.**
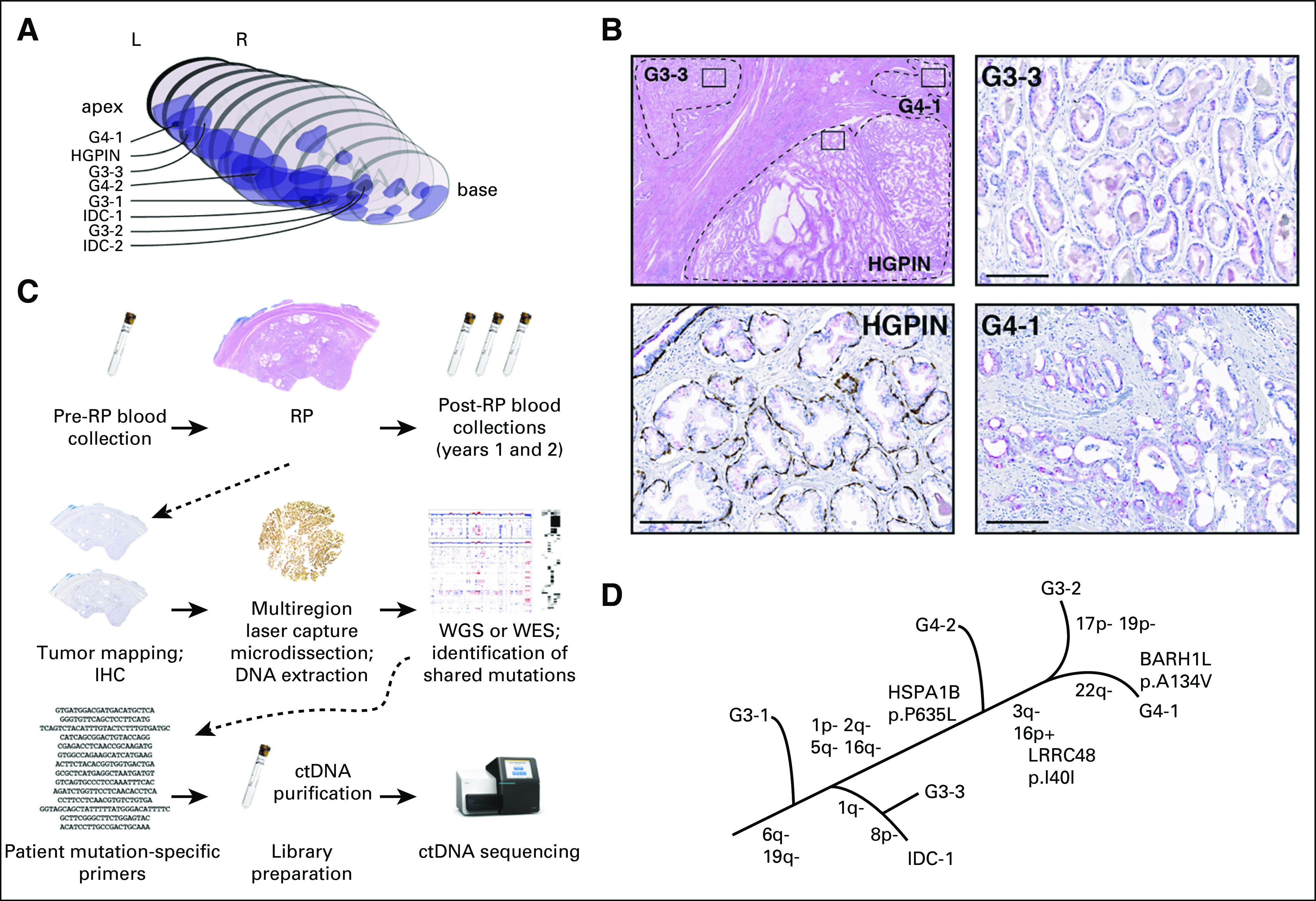
Multiregion sampling of prostate cancer tissue for identification of alleles to be detected in circulating tumor DNA (ctDNA). (A) Representative case demonstrating mapping of tumor throughout the prostate and the selection of distinct histologies that may represent major branches of the tumor system. (B) Hematoxylin and eosin staining and PIN-4 cocktail immunostaining of three adjacent histologies. Brown chromogen: p63 and cytokeratins 5 and 14; red chromogen: α-methylacyl coenzyme A racemase; scale bar: 200 μm. (C) General schematic of workflow showing the sequencing of patient tissue for identifying candidate alleles and the retrospective analysis of those alleles in banked plasma from the same patient. (D) Phylogenetic tree of tumor foci from the prostate cancer mapped in (A). Although copy number alterations and point mutations are used for establishing the evolutionary tree, only point mutations are sequenced in plasma specimens. IHC, immunohistochemistry; RP, radical prostatectomy; WES, whole-exome sequencing; WGS, whole-genome sequencing.

When mapped, prostate cancers branch substantially at their index lesion (Fig 2A), such that repeated sampling of multiple tumor regions (Fig 2B) is needed to empirically infer mutations that are shared by all or most tumor lesions, and thus would be candidates for detection in ctDNA. Our approach attempts to identify such mutations through several steps, as illustrated in Fig 2C: (1) immunohistochemistry was performed on serial sections of multiple blocks of tumor tissue from each patient; (2) laser capture microdissection was used to isolate histologically distinct foci from which DNA was extracted; (3) extracted DNA was subjected to WGS and whole-exome sequencing (WES); and (4) WGS and WES data were integrated into tumor phylogenies encompassing SCNAs and point mutations (Fig 2D). Point mutations composing the “trunk” or major “branches” of these evolutionary “trees” were selected for incorporation into the patient-specific assay.

### Single Molecule Detection

Discordance between different commercial tests and even repetitions of standard polymerase chain reaction (PCR) to detect and quantify rare alleles can often be linked to high false-positive rates.^26,27^ Consequently, we designed a locus-specific, Illumina-compatible (San Diego, CA) library design that incorporated 7-base unique molecular identifiers for tagging individual template molecules. Coupled with analysis scripts that use heuristics, this design distinguished mutations arising from errors during library preparation from those present in the starting material, making it robust to false-positive results. Details of library design and analysis are provided in the Appendix.

To verify and benchmark this design, we first generated target amplicons of 139 or fewer base pairs (Data Supplement), containing eight different heterozygous and homozygous alleles from PC3 and DU145 genomic DNA (gDNA), to serve as synthetic ctDNA. Fresh plasma was obtained from a single male donor with no known cancer diagnoses, through the National Institutes of Health Department of Transfusion Medicine. Synthetic ctDNA was spiked into separate 3-mL aliquots of plasma in duplicate for both DU145- and PC3-derived gDNA in approximate copy number amounts spanning six logs (ie, 10^1^, 10^2^, 10^3^, 10^4^, 10^5^, and 10^6^) for a total of 24 plasma samples plus four negative controls. Samples were frozen overnight and later thawed for extraction of cfDNA. Two rounds of library preparation were performed per cfDNA sample, for a total of 56 libraries.

As shown in the Data Supplement, the assay demonstrated high reproducibility between spike-in targets at similar quantities, with robust detection of mutant alleles at the 10 and 100 spike-in amounts. Although the expected limit of detection on the basis of the total possible number of unique molecular identifiers was 16,384 (ie, 4^7^) template molecules, our observed mean saturation was closer to 1,000 molecules (Data Supplement), as a result of the abundance of the wild-type allele, which our analysis ignored. Although we spiked in excess quantities of target for the purpose of estimating recovery, yield, and complexity loss during library preparation, this assay demonstrated robust recovery of rare alleles, which was its intended purpose.

### Positive Detection of ctDNA Alleles in Plasma From Patients With Metastatic Prostate Cancer

**FIG 3.**
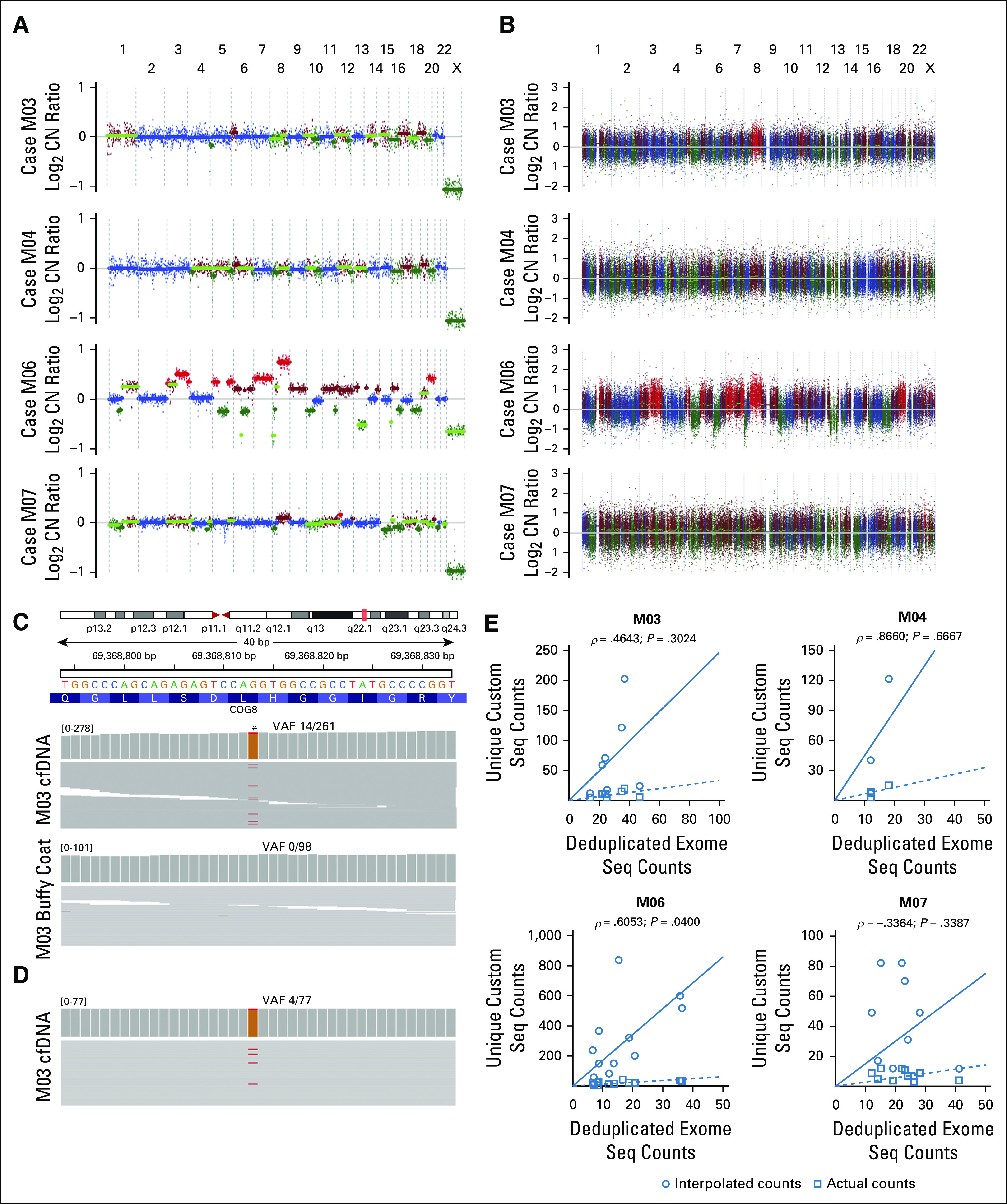
Detection of circulating tumor DNA in plasma from four patients with metastatic prostate cancer. (A, B) Plots of log_2_ copy number (CN) ratio (cfDNA *v* buffy coat) for patients M03, M04, M06, and M07 as determined by (A) ultra-low-pass whole-genome sequencing and (B) high-depth whole-exome sequencing (WES). (C) A representative somatic point mutation (g.chr16:69368813G>T) observed in the cfDNA from patient M03 by WES. (D) Representative bespoke sequencing detection of mutant g.chr16:69368813G>T allele. (E) Scatter plots showing relationship of actual and adjusted (interpolated) bespoke sequencing mutant read counts versus mutant read counts from exome sequencing. Correlation statistic Spearman ρ and *P* values are the same for actual and interpolated counts. cfDNA, cell-free DNA; Seq, sequencing; VAF, variant allele fraction (mutant reads/total reads). (*) Mutant allele.

Tissue biopsy specimens from four of seven patients with metastatic cancer (M03, M04, M06, and M07) with high plasma tumor content, as determined by ULP-WGS (Fig 1), were unavailable for sequencing. WES was performed on cfDNA from these four cases, using their matched buffy coat gDNA as a benign control. cfDNA and buffy coat DNA were sequenced to mean on-target depths of 140× and 90×, respectively. As expected, the SCNA profile from exome sequencing generally matched the SCNA profile from ULP-WGS for each patient (Fig 3A), although the substantially higher resolution of exome sequencing permitted detection of smaller genomic events (Fig 3B).

Importantly, for samples with orthogonally confirmed ctDNA levels, we generated a catalog of somatic mutations by comparing each sample with its matched benign control (Fig 3C; Data Supplement). Following our protocol for bespoke library design, four sets of primers were generated for the detection of high-clonality mutant alleles in each sample (Data Supplement). These primer pairs successfully amplified 36 of 36 targets from all four cases and detected mutant ctDNA alleles in 32 of 36 amplified targets (Fig 3D; Data Supplement). When back calculated to an expected molecule number using the spike-in curve fit, raw deduplicated mutant read counts from the patient-specific assay correlated well with the unique mutant read counts from exome sequencing (Fig 3E).

Detection of mutant alleles in cfDNA was also positively correlated with raw read count abundance (Spearman’s ρ = 0.5575; *P* < .001; Data Supplement). Although interpolation of actual read counts to estimate the number of starting template molecules generally increased the absolute number of alleles reported, the interpolated values were more similar in range to the number of deduplicated, exome-sequenced detected alleles than the actual count (Wilcoxon matched-pairs signed rank test; Data Supplement).

We next asked whether lower read count thresholds would affect binary detection (presence or absence) of ctDNA below defined read-count thresholds. When sequence reads were downsampled before alignment to 1 million reads per library, or 100,000, 50,000, or 10,000 reads per target, the observed read count was consistently higher than what would be expected, because of lower depth (Data Supplement), such that reduction to 10,000 reads per target (greater than 90% downsampling) only reduced mutant allele detection by approximately 50%. At the lowest level of downsampling (10,000 reads per target), all mutant alleles were still detected. Importantly, at sites where mutations were not previously detected, analysis of full-sequence output did not reveal mutations that would represent artifacts of library preparation or sequencing. Therefore, from these data, we conclude that our patient-specific assay can robustly resequence mutant cfDNA alleles with greater than 93% sensitivity and 100% specificity for the target regions assessed.

### Lack of Detection of ctDNA Alleles in Plasma From Patients With Localized Prostate Cancer

With a highly sensitive patient-specific assay robust against false-positive results, we applied our ctDNA detection approach to men newly diagnosed with localized disease. Our initial hypothesis was that detection of ctDNA at baseline would predict adverse pathologic features associated with recurrence (such as high Gleason score) or would predict recurrence itself. We selected nine of the 112 localized disease cases, representing a range of Gleason scores, pathologic T stages, sample ages, baseline PSA levels, and biochemical recurrence statuses (3 years or more after prostatectomy), from which to identify clonal markers in plasma (Table 2). A list of microdissected and sequenced foci is given in the Data Supplement.

**TABLE 2.**
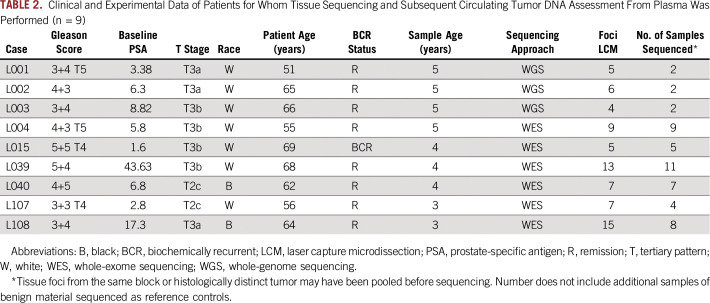
Clinical and Experimental Data of Patients for Whom Tissue Sequencing and Subsequent Circulating Tumor DNA Assessment From Plasma Was Performed (n = 9)

Before laser capture microdissection, we performed immunohistochemistry against ERG to select concordantly positive or negative foci (Data Supplement). We previously established that chromosomal breakpoints serve as a definitive clonal marker in TMPRSS2:ERG fusion-positive tumors.^28^ In only one (L001) of two ERG-positive cases (L001 and L003) for which WGS was performed did we successfully read through the *TMPRSS2-ERG* breakpoint (Data Supplement). However, even a nested PCR approach failed to amplify the fragment of DNA containing the breakpoint from plasma (Data Supplement).

Therefore, we used the approach illustrated in Figure 2, in which we integrated SCNA and mutation clonality data from multiple foci (Data Supplement) to identify point mutations as either trunks, branches, or leaves of a given tumor tree; truncal mutations were shared by all foci, branch mutations were shared by most or some foci, and leaf mutations were unique to a given focus. The complete list of somatic mutations considered for this analysis and the mutations selected for bespoke sequencing ctDNA analysis are given in the Data Supplement. Despite high specificity and coverage, no mutated alleles indicative of ctDNA were detected from the cfDNA sampled before RP (Table 3). Surprisingly, any ctDNA that may have been present from patient L015, who had Gleason 10 prostate cancer and subsequently had disease recurrence, was below the limit of detection for both ULP-WGS (Fig 1) and allele-specific measurement (Table 3; Data Supplement).

**TABLE 3.**
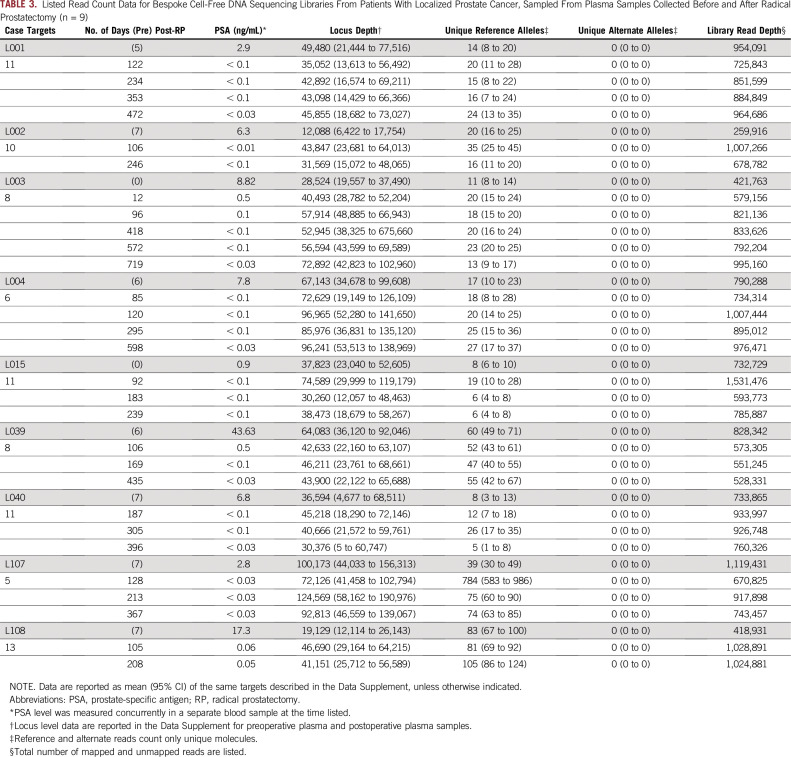
Listed Read Count Data for Bespoke Cell-Free DNA Sequencing Libraries From Patients With Localized Prostate Cancer, Sampled From Plasma Samples Collected Before and After Radical Prostatectomy (n = 9)

Finally, we asked whether we could detect ctDNA in this same group of patients after RP when PSA levels are low before biochemical recurrence. Although only one patient in this cohort has had disease recurrence to date, ctDNA was not detected in any of the nine patients over multiple time points (Table 3; Data Supplement). Taken together, we conclude that although allele-specific detection is a robust approach for identifying ctDNA alleles in patients with metastatic prostate cancer, it is inferior to the sensitivity of PSA testing in a localized prostate cancer population for measuring disease burden.

### Data Availability

Sequence data has been deposited into dbGaP (accession ID phs001813.v1.p1).

## DISCUSSION

In light of concerns that PSA levels simply reflect tumor volume rather than grade, and that they may fail to detect androgen receptor–low or indifferent tumors, PSA measurement remains an excellent biomarker for treatment response, and it is the gold standard for diagnosing biochemical recurrence after primary therapy.^29^ In our study, we hypothesized that higher grade, more poorly differentiated cancers could be distinguished from indolent tumors on the basis of detection of ctDNA in preoperative plasma. We also hypothesized that plasma from patients with more aggressive tumors that ultimately recurred would also harbor ctDNA that could be detected preoperatively, or postoperatively before biochemical recurrence defined by PSA level. Using unbiased ULP-WGS, we were unable to detect ctDNA in localized prostate cancer before surgery from patients with a wide range of PSA levels and tumor aggressiveness. Our allele-specific assay, which is sensitive to as few as 10 mutant alleles of spiked-in DNA, similarly did not detect either ctDNA from preoperative plasma or from plasma before biochemical recurrence. In contrast, both assays detected ctDNA in patients with metastatic prostate cancer.

If ctDNA levels were directly proportional to PSA levels, then a subset of patients with localized prostate cancer with higher PSA levels would have been expected to have detectable ctDNA.^30^ Indeed, in our metastatic cohort, three patients with PSA levels below 30 ng/mL did not show any SCNAs by ULP-WGS, with the remainder (including one patient with PSA level of 51.34 ng/mL) having ctDNA detectable by ULP-WGS, WES, and allele-specific sequencing. However, among the localized cohort, even the patient with the highest preoperative PSA level (L039; 43.63 ng/mL) did not harbor detectable ctDNA. This finding suggests that intrinsic differences between primary and metastatic prostate cancer, including the kinetics of ctDNA shedding and turnover, low proliferative rate of localized disease, and poor proximity to vasculature relative to metastases, may result in degradation of cfDNA before it reaches circulation.

Because ctDNA potentially represents a pool of multiple subclones shedding cfDNA, alleles detected in ctDNA may only represent the most clonal and truncal of alterations, especially when the percentage of ctDNA in total cfDNA is low.^16^ To address this challenge, we reconstructed tumor phylogenies from genome and exome sequencing of tissue to select alleles representing the major subclones that would be present at the time of surgery and further mediate relapse. Before executing these experiments, we developed and tested a patient-specific, allele-specific sequencing assay that satisfied requirements for reproducibility, accuracy, sensitivity, and specificity.^16,31,32^ This assay consistently detected spiked-in alleles and showed 100% concordance to unbiased WES of the same sample at very high coverage. However, after applying this assay to preoperative and postoperative plasma samples, we found that the lack of detection of clonal alleles in ctDNA was not predictive of adverse final pathology, recurrence, or metastasis.

There is an important limitation of this finding. Although rare, some prostate cancer tumors recur that were only a minor subclone at the time of RP.^33^ In our study, we focused on the index lesion as the tumor system most likely to drive relapse. Given the prospective and unselected population of our cohort, the vast majority have remained in remission after surgery, with the only one recurrent tumor (patient L015) having undergone in-depth primary tumor sequencing. Moreover, we were unable to acquire metastatic tissue from this patient to sequence and compare with the targets selected from the prostatectomy. Consequently, it is possible that the clone driving metastasis was independent of the tissue sequenced. Despite using the most sensitive allele-specific assay possible, design of these assays was based on comprehensive tumor sampling. Therefore, we cannot state with absolute certainty that allele-specific analysis assessed the correct clone and that ctDNA levels in patients were below limits of detection.

There have been a limited number of published studies that evaluated cfDNA as a biomarker prognostic of advanced disease in the localized prostate cancer setting.^34-36^ The largest of these studies to date examined the total burden of cfDNA and ctDNA by hypermethylation of the *GSTP1* promoter in DNA extracted from the serum of 192 patients.^35^ Although *GSTP1* hypermethylation in serum cfDNA was increased in the recurrent and metastatic populations compared with indolent prostate cancer, contribution of *GSTP1* equivalents in serum from normal tissue affected by oligometastases may have contributed to this finding, because *GSTP1* hypermethylation is not a tumor-specific event.^35^ Moreover, the PCR assay used for detecting circulating *GSTP1* amplifies a DNA fragment in excess of the approximately 165-bp ctDNA fragment, suggesting it is of nontumor origin despite reflecting increased tumor aggressiveness.^35^

Bespoke approaches to detect ctDNA from urothelial and colorectal cancers have demonstrated success in risk stratification and therapy monitoring. In a cohort of 68 patients with muscle invasive bladder cancer, a personalized assay to sequence somatic variants as markers of ctDNA in preoperative plasma was highly prognostic for recurrence after cystectomy.^37^ Among patients with recurrent disease, ctDNA detected before chemotherapy also tracked with worse overall survival.^37^ Similar successes were achieved in a cohort of 130 patients with colorectal cancer: A personalized ctDNA detection assay detected ctDNA in 88.5% of preoperative plasma samples, and 70% of patients with detectable ctDNA at the start of adjuvant chemotherapy subsequently experienced disease recurrence.^21^ The striking difference between our findings and these reports from bladder and colorectal cancer cohorts may reflect some of the same differences between primary and metastatic prostate cancer with respect to ctDNA shedding, including cell proliferation rate and proximity to vasculature.

Nonetheless, to the best of our knowledge, this is the first comprehensive analysis to conclude definitively that somatic mutation and copy number alterations in cfDNA do not effectively measure ctDNA levels in an untreated localized prostate cancer cohort. Because these locus-level analyses of individual genomes are below the limits of detection, other circulating nucleic acid analytes may be more representative of phenotype and thus offer better detection characteristics. Circulating tumor cells, circulating cell-free microRNA, circular RNA, post-transcriptionally modified RNA species, and genome-wide tissue-of-origin patterns of DNA methylation do not correlate 1:1 with tumor cell number, and thus may give a much greater signal than allele-dependent assays for the early, noninvasive detection of aggressive and potentially recurrent prostate cancer.^37,38^

## APPENDIX MATERIALS AND METHODS

### Study Approval

This research was conducted in accordance with the principles of the Declaration of Helsinki. The collection and analysis of plasma, tissue, and demographic data from patients with localized prostate cancer was approved by the institutional review boards of Beth Israel Deaconess Medical Center (protocol no. 2010-P-000254/0) and Dana-Farber/Harvard Cancer Center (DFHCC; protocol no. 15-008 and 15-492). The collection of plasma and demographic data from patients with metastatic prostate cancer was approved by the National Institutes of Health Institutional Review Board (protocol no. 02-c-0179). All patients provided informed consent before participating in tissue procurement protocols.

### Blood Collection

Blood was obtained from patients diagnosed with localized prostate cancer pre- or perioperatively to radical prostatectomy and again at intervals coinciding with urology follow-up visits. At each time point, 8 to 10 mL of whole blood was collected into K_2_-EDTA Vacutainer (Becton Dickinson, Franklin Lakes, NJ) or Cell-Free DNA (cfDNA) blood collection tubes (BCTs; Streck, La Vista, NE) following recommended guidelines for venipuncture, collection order, and inversion. K_2_-EDTA–collected blood was stored on ice and processed within 1 hour of collection. cfDNA BCT-collected blood was stored at room temperature and processed within 3 days of collection (if shipped from DFHCC to the National Institutes of Health) or within 8 hours of collection (if processed at DFHCC).

Blood was obtained from patients with metastatic, castration-resistant prostate cancer after progression while being treated. At a single time point before receiving subsequent therapy, 8 to 10 mL of whole blood was collected into cfDNA BCTs as described. Sample processing occurred within 8 hours of collection.

### Tissue Histology

Radical prostatectomy specimens were grossly examined and then formalin-fixed and paraffin-embedded according to standard procedures. Hematoxylin-and-eosin–stained slides were reviewed by a board-certified surgical pathologist following the 2014 International Society of Urological Pathology guidelines. For each case, maps were created to identify the distribution of cancerous regions throughout the entire resected specimen.

### Laser Capture Microdissection

Serial sections of tumor tissue (and benign regions uninvolved with tumor) were cut onto metal frame PEN-membrane slides (MicroDissect, Herborn, Germany), stained with Paradise Stain (Thermo Fisher Scientific, Waltham, MA), and laser capture microdissected using an ArcturusXT Ti microscope (Thermo Fisher Scientific) onto CapSure Macro LCM Caps (Thermo Fisher Scientific). Slides were stained with hematoxylin and eosin, ERG, PTEN, and PIN-4, scanned and visualized using ZEN Browser (Carl Zeiss, Oberkochen, Germany) on an adjacent monitor as references. Per focus, 50,000 to 100,000 cells were captured. For each cap, a photomicrograph was acquired to estimate tumor cell purity in each sample.

### Whole-Genome and Whole-Exome Sequencing of Tissue DNA

Genomic DNA (gDNA) was sheared using acoustic sonication (Covaris, Woburn, MA). For whole-genome sequencing, target genomic fragment sizes were longer than 300 bp and selected by Pippin Prep (Sage Science, Beverly, MA). After end-repair and A-tailing, modified Illumina adaptors containing 7-base inline unique molecular identifiers at the 3′ end of each adaptor were ligated to each library insert. Libraries were sequenced on a HiSeq X10 (Illumina, San Diego, CA) to a target depth of 30× to 40× coverage.

For whole-exome sequencing (WES), target fragments were sonicated to a target size of 200 bp and selected by AMPure XP SPRI beads (Danaher, Washington, DC). WES libraries were prepared using the SeqCap EZ Exome Kit v3 (Roche, Basel, Switzerland) or the SureSelect Human All Exon V7 Low Input Exome kit (Agilent, Santa Clara, CA). Equimolar pooled libraries were sequenced on a HiSeq 2000 and 4000 (Illumina) to a targeted on-bait depth of 150×. Agilent libraries included an additional R3 read to sequence the 10-base unique molecular identifier.

### Ultra-Low-Pass Whole-Genome Sequencing of Plasma DNA

From 10 to 100 ng of cfDNA from plasma samples (corresponding to approximately 10 μL of eluate) or 100 ng of gDNA was assembled into paired-end libraries using the NEBNext Ultra II DNA Library Prep Kit (New England BioLabs, Ipswich, MA). Approximately 60 libraries were pooled per lane before sequencing on a HiSeq 4000 to a target depth of 0.5×.

### WES of Plasma DNA

From 10 to 100 ng of cfDNA from plasma samples (corresponding to approximately 10 μL of eluate) or 100 ng of gDNA was assembled into exome sequencing libraries using the SureSelect Human All Exon V7 Exome kit (Agilent). DNA samples were pooled to achieve an on-bait depth of 100× for the buffy coat gDNA and 300× for the plasma cfDNA.

### Statistical Analyses

Statistical analyses were performed using GraphPad Prism 8 for Mac. Statistical tests used and relevant variables are indicated in the legend of each figure.
